# Application of Hierarchical Clustering to Analyze Solvent-Accessible Surface Area Patterns in *Amycolatopsis lipases*

**DOI:** 10.3390/biology11050652

**Published:** 2022-04-24

**Authors:** Supajit Sraphet, Bagher Javadi

**Affiliations:** 1 Institute of Molecular Biosciences, Mahidol University, Nakhon Pathom 73170, Thailand; supajit.sra@mahidol.ac.th; 2 Department of Sciences, Faculty of Science and Technology, Suan Sunandha Rajabhat University, Bangkok 10300, Thailand

**Keywords:** *Amycolatopsis eburnea*, conserved accessible solvent area, enzyme, lipase, protein stability, structural biology analysis

## Abstract

**Simple Summary:**

Solvent-Accessible Surface Area (SASA) as the one dimensional structure property of the protein considers as the measuring the exposure of an amino acid residue to the solvent in one protein. It is an important structural property as the active sites of proteins are mostly located on the protein surfaces. The aim of this paper is to provide the clear information on different *Amycolatopsis eburnea* lipases based on the SASA patterns. This information could help in recognizing the structural stability and conformation as well as precise clustering them for revealing lipase evolution.

**Abstract:**

The wealth of biological databases provides a valuable asset to understand evolution at a molecular level. This research presents the machine learning approach, an unsupervised agglomerative hierarchical clustering analysis of invariant solvent accessible surface areas and conserved structural features of *Amycolatopsis eburnea* lipases to exploit the enzyme stability and evolution. *Amycolatopsis eburnea* lipase sequences were retrieved from biological database. Six structural conserved regions and their residues were identified. Total Solvent Accessible Surface Area (SASA) and structural conserved-SASA with unsupervised agglomerative hierarchical algorithm were clustered lipases in three distinct groups (99/96%). The minimum SASA of nucleus residues was related to Lipase-4. It is clearly shown that the overall side chain of SASA was higher than the backbone in all enzymes. The SASA pattern of conserved regions clearly showed the evolutionary conservation areas that stabilized *Amycolatopsis eburnea* lipase structures. This research can bring new insight in protein design based on structurally conserved SASA in lipases with the help of a machine learning approach.

## 1. Introduction

Hydrophobic forces in proteins play a vital role in the stability, folding and protein–protein interaction [1,2]. The residues comprised in hydrophobic areas, their interactions and the form packing could be useful for studying the protein structure and protein-substrate binding [3,4]. The residues involved in core stability of proteins are hydrophobic residues. Therefore, finding protein and enzyme Solvent Accessible Surface Area (SASA) [5,6] and hydrophobic areas of total and conserved residues and clustering them could provide unique features in comparing the proteins and enzymes [7]. Furthermore, these residues, their interaction and classification could extrapolate the protein contact map by emphasizing the role of each specific residue in protein stability and conformation [8]. This paper provides insight on SASA patterns in *Amycolatopsis eburnea* lipases and clustering of conserved structural-SASA with the help of the unsupervised agglomerative hierarchical method (machine learning approach) toward identification of hot spot structures for protein stability and conformation. The results will help in the design and engineering of new enzymes.

Glycerol ester hydrolase or triacylglycerol acylhydrolase is (E.C.3.1.1.3), a fat splitting enzyme which is also called Lipase [9]. The products of the enzymatic reaction (as a catalyzer) (hydrolyses triglycerides) are glycerol and fatty acids. Applications of lipase include food, dairy, flavor, pharmaceuticals, biofuels, leather, cosmetics, detergent, and many chemical industries [10,11,12]. This is the third most significant enzyme in the industry after proteases and amylases [13]. This enzyme can hydrolyze triglycerides in both aqueous and non-aqueous media [14,15]. It should be mentioned here that lipase substrates are insoluble in water. Lipase loses its functionality in different organic solvents [16]. Structurally, lipases contain a/b hydrolase folds [17]. Triad of Ser, Asp (Glu) and His residues in their active site were considered as their specific structural characterization [18,19].

It is clearly established that around seventy medicinal and agricultural microbial products are from the Actinobacteria phylum. The famous ones are different antibiotics produced by the *Amycolatopsis* genus [20,21]. This genus is very remarkable in producing antibiotics such as balhimycin, vancomycin, as well as immuno-suppressants, anti-cancer agents and many other secondary metabolites [22,23,24,25]. Their significant position in the medicine and agriculture market is due to the diversity of vital compounds and the amount of production [21]. This substantial position, besides the innumerable structure in their compound and eventually their genomes, provides a great prospect for researchers to discover valuable insight for future applications. Therefore, *Amycolatopsis* species require study to find the details of structure and diversity of important enzymes such as lipases [26,27,28,29,30]. It was recently reported that *Amycolatopsis eburnea,* one of the species in the *Amycolatopsis* genus, has a symbiotic relationship with mycorrhizal fungi; however, the details of this mechanism need to be investigated.

Computational characterizations of enzymes with the help of machine learning algorithms offer a great opportunity to speed up the systematic classifications [31,32]. They can help routine scientific proposals to engineer better enzymes with superior activity. Additionally, computational analysis offers a clearer way to understand the mechanisms of each reaction from a structural point of view. Lipase enzymes with very wide applications can get much more benefit from these computational approaches [33]. Moreover, designing the most efficient experiments in the lab requires clear computational and structural information [31,33].

The lipases with microbial origin can be isolated from different cellular compartments, either extracellular, peripheral protein or intracellular enzymes [34,35,36]. It is accepted that structural features of enzymes are the key indicators for protein evolution despite the sequence differences. The folding and stability of proteins in general and enzymes in particular are highly dependent to their structure and environment. The structural plasticity of enzymes in different environments is the key to functionality efficiency. As no significant sequence similarity was observed in the conserved folding in many enzymes, the regions with particular structure that were conserved in the enzyme would play a critical role for plasticity and eventually functionality. On the other hand, not all residues of enzymes were involved in determination of folding and stability. For detecting the sequence necessity for particular fold and stability and their role in enzyme evolution, Solvent Accessible Surface Area was applied. This feature is based on the fact that hydrophobic residues have less/no SASA [37].

The lack of information on structural stability of lipase from the *Amycolatopsis eburnea* with their inevitable place in industry is noticeable. Therefore, the aim of this research paper is to provide insight and clear information on different *Amycolatopsis eburnea* lipases based on the SASA. This information can help in recognizing the lipases structural stability and conformation. The clear information of each amino acid in the structure could help in designing the new lipase enzyme with better functionality. Furthermore, this precise clustering of specific amino acids can demystify the lipase evolution and even enzyme functionality.

## 2. Methods

The sequence data were retrieved from the National Center for Biotechnology Information (NCBI) database for *Amycolatopsis eburnea* lipases. The physiochemical parameters and 3-D homology models were calculated with the help of bioinformatics-server (https://www.expasy.org/) (accessed on 1 February 2020) [38]. Furthermore, for confirmation and comparison of 3D-models, the deep learning de novo modeling was performed for all lipases. In this method, after generating the multiple sequence alignment, the prediction for distance and orientation distribution was done, followed by coarse grained structure modeling by energy minimization, full atom structure refinement and finally generating the models. The percentage of similarity and structural identity were also calculated for all models [39]. The phylogenetic relationship of sequences were presented with MegaX software [40]. The secondary structure predictions were performed with the help of Chou & Fasman secondary structure prediction [41]. The clustering of SASA determined with the hierarchical clustering method that groups together the more close or similar SASA. In this paper, the agglomerative approach which the bottom-up of each SASA data (as the observation) was considered as one cluster and merged with the closer cluster as one moved up the hierarchy. In order to calculate the distance between the SASA (proteins), Euclidean distance and Ward method were applied.

### Solvent Accessible Surface Area (SASA)

The solvent accessible surface area of each enzyme was calculated according to Fraczkiewicz and Braun [42]. The Cartesian coordinates of protein atoms stored in PDB models were used to calculate the SASA for each residue [43]. The solvent associable surface area of residues was calculated for two environments: the nucleus and surface for each enzyme. The area contacts between solvent and the atoms as the points located on a sphere interaction radius surrounding them were identified as SASA. For this calculation, the interaction is the coverage of van der Waals radius of each atom type, plus the radius of a water molecule. The individual protein chain and the similar coverage of each enzymes area were calculated and compared. For categorizing the residues of proteins as nucleus or surface, the side-chain solvent surface accessibility is divided by the specific accessibility value for each residue. The specific accessibility value is the average solvent accessible surface area in the tripeptide Gly-X-Gly in an ensemble of 30 random conformations. Thus, residues with ratio value more than 50% were considered as in surface environment and alternatively the residues with less than 20% marked as nucleus or core environment.

$$Total\;average\;SASA=\frac{Nucleus\;SASA+Surface\;SASA}{Total\;amino\;acids}$$

Structurally conserved regions (SCR) for each enzyme were identified with the help of Chimera with defaults parameters [44] and solvent accessible surface areas of each SCR were calculated as mentioned above. The SCR-SASA is herein considered as the new conserved fingerprint descriptor for *Amycolatopsis eburnea* lipases. The cluster analyses of SASA patterns of lipases were performed with unsupervised agglomerative hierarchical clustering method as a machine learning approach with the help of python 3.9 programming language (http://www.python.org) (accessed on 21 August 2021).

## 3. Results

Physiochemical features of *Amycolatopsis eburnea* lipases showed in Table 1. The numbers of amino acids for lipases were in the range of 252 to 436. The average molecular weight was around 38 kDa. The minimum and maximum of MW were 24 kDa and 44 kDa, respectively. The negatively charged amino acid were in the range of 19 to 47. However, the positively charged amino acids were lower than them. The pI of lipases was more than 4.52 with a maximum of 6.23. This showed that all of them were in the range of acidic pH condition. Therefore, we need to find out if these enzymes performed their function in acidic environments. Furthermore, buffer preparation for purifying them should receive great attention with these value indexes. The aliphatic indexes (thermal stability of enzymes) were in the range of 73 to 97. The hydrophobicity of lipases presented with the Grand Average of hydropathicity (GRAVY) values in the range of −0.110 to 0.280. The average of GRAVY was 0.7.

**Figure 1:**
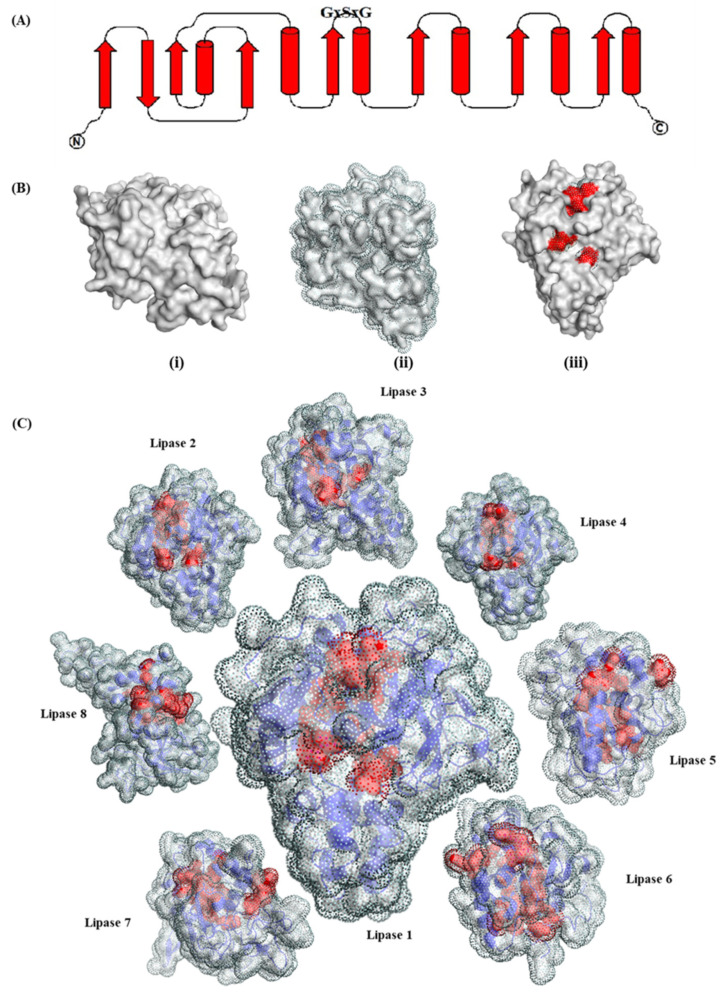
Lipase topology diagram with the strands indicated by arrow and helices by cylinder (**A**) Surface model representation of *Amycolatopsis eburnea* lipase 1 alone. (i) total SASA (ii) and Structurally conserved SASA in red (iii) (**B**) *Amycolatopsis eburnea* lipases modelled with deep learning de novo (**C**) (structurally conserved SASA in red, ribbon representation of lipases structures in blue).

The three-dimensional structure of the enzymes was modeled with homology modeling with the help of Swiss institute of bioinformatics-server (Figure 1). All models were then evaluated for stereo chemical quality with Ramachandran Map (Ramachandran and Sasisekharan 1968), as well as qmean for model confirmation (Table 2 and Table 3). *Amycolatopsis eburnea* lipase sequences were again modeled with deep learning de novo modeling as described by Yang and coworkers [39]. The results (models) provided by de novo modeling were also confirmed with Ramachandran and qmean methods. The quality of models significantly improved (Table 3). Ramachandran map showed that less than 1.92% (A0A3R9DV90) of residues were in the outlier section; thus, the models are fully acceptable. The favorite region residues were more than 91% which showed the high quality of modeling in comparison to homology modeling performed earlier. The information provided confirmed the models for further analysis. All lipase models were homodimer. Ramachandran results showed that maximum residues in the favored region were 96.85% (A0A3R9EQB2). The indexes for qmean were more than −1.98 and considered acceptable for all models (de novo).

The *Amycolatopsis eburnea* lipases showed less frequency of His, Met and Cys and Trp compared to other residues (Table 4). The secondary structure in lipases is shown in Table 5. The percentages of helices in the structure of lipases were higher than beta sheets and turn loops. At least 53.7% (A0A3R9DV90) of the lipases structure was helices.

**Figure 2:**
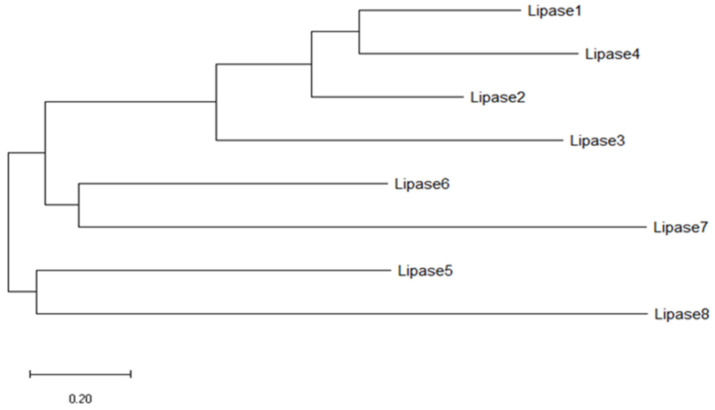
Dendrogram of lipase enzyme sequences.

**Figure 3:**
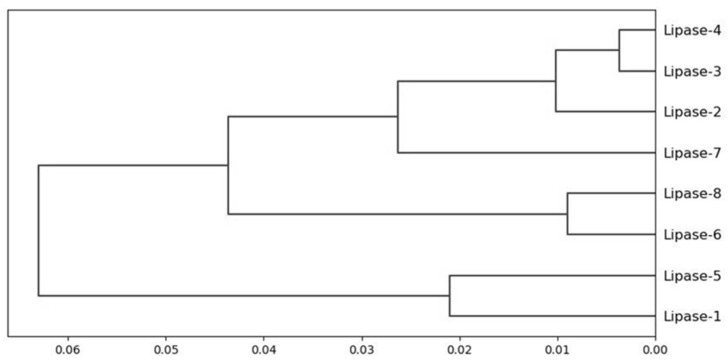
The hierarchical clustering of *Amycolatopsis eburnea* lipases is based on solvent surface accessibility area.

The total SASA of the enzymes was applied to cluster the *Amycolatopsis eburnea* lipases. The overall similarity of lipases was 99.96%. The dendogram result showed lipase 4 and lipase 3, with lipase 2 had more than 99.99% similarity. This similarity percentage was also observed with lipase 6 and lipase 8. However, the similarity of these two mentioned clusters was around 99.94%. Three distinct clusters were observed and categorized the lipases overall. A clear identification of SASA clustering is one of the great advantages of this grouping even in lipases with very high sequence similarity (Figure 2 and Figure 3).

The total solvent accessible surface area and average of solvent accessible for two environments (nucleus and surface) in each enzyme is shown in Table 6. The maximum SASA of nucleus residues was related to lipase 4; however, the maximum SASA of surface residues was related to lipase 8. The average solvent accessibility areas of enzymes were between 39.65 to 51.53 Å^2^. The overall side chain of solvent accessibility areas both in the nucleus and surface environments were higher than backbones. This trend was observed in individual enzymes as well. The results showed that lipase 4 had more of a chance to interact with solvent. Furthermore, results showed more accessibility for side chains of the enzymes to interact with solvent and eventually substrate compared to enzyme backbones.

**Figure 4:**
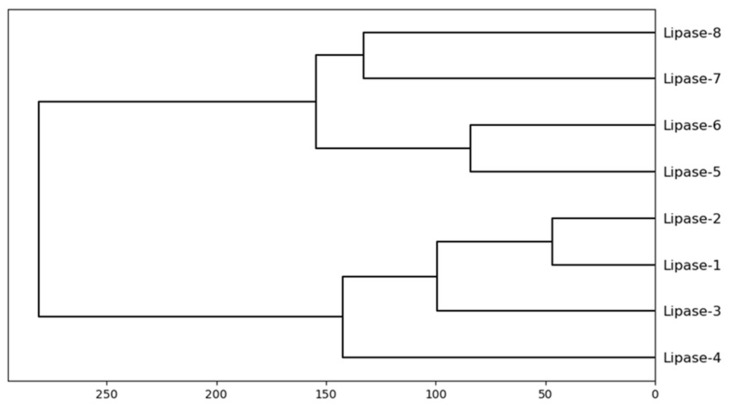
Hierarchical clustering of structurally conserved regions-SASA.

Hierarchical clustering of structurally conserved regions-SASA of *Amycolatopsis eburnea* lipases is shown in Figure 4. Lipases 1, lipase 2, lipase 3, and lipase 4 showed the similarity approximately the same as the lipase 5, lipase 6, lipase 7, and lipase 8. Lipase 1 and lipase 2 with the minimum dissimilarity showed the more conserved SASA compared to other lipases. On the other hand, the lipase 8 and lipase 7 are totally different compared to lipase 3 or lipase 4. Overall, dendogram showed more clear similarity features compared to the whole enzyme SASA. The structurally conserved regions-SASA could provide more flexibility to select the lipase for specific substrate based on the contact area to the solvent.

**Figure 5:**
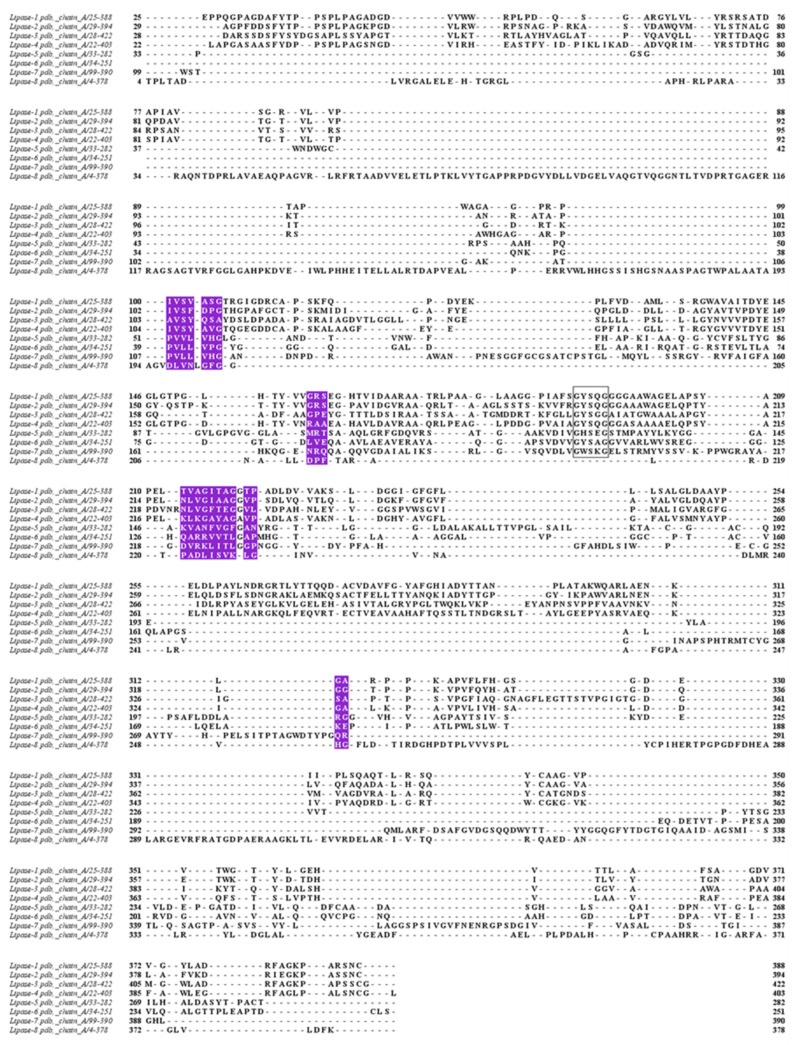
The proteins sequences were aligned using Chimera with defaults parameters. Structurally conserved regions are shown in blue.

The structurally conserved regions (Figure 5) showed the correlation in the SASA (Table 7). Lipase 5 and lipase 6 had the highest correlation, followed by lipase 1 and lipase 2; however, the residues involving the structure were not the same. The lowest SASA correlation was related to lipase 5 and lipase 3, followed by lipase 4 and lipase 7. The SASA correlation of different structurally conserved regions showed the overall high correlation between *Amycolatopsis eburnea* lipases. The conserved regions SASA might indicate the minimum SASA which was essential for stability of the protein and folding.

**Figure 6:**
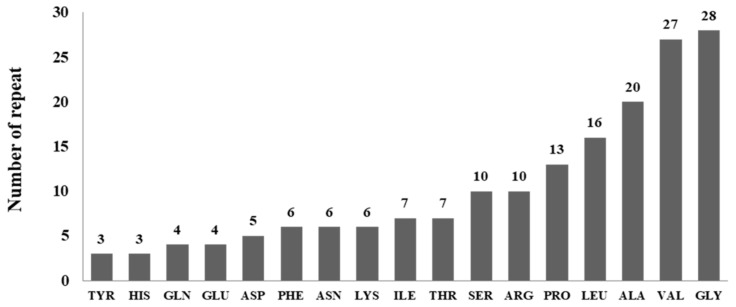
Residues preferences of conserved regions.

The similarity of SASA in conserved regions could shed light on the conserved and preferences of residues for the stability of *Amycolatopsis eburnea* lipases. It was observed that GLY and Val are the most frequent residues in conserved regions with 28 and 27 repeats. Different residues were shown in Figure 6. Three residues of CYS, MET and TRP were not observed in the structurally conserved region of *Amycolatopsis eburnea* lipases.

## 4. Discussion

There is an increased concern for lipase as the third most important enzyme in the market for hydrolyzing triglycerides in different media [14,19,45,46,47,48]. The enzyme markets are food, dairy, flavor, detergent, pharmaceuticals, biofuels and cosmetics industries. The demand of more than 1000 tons of lipases for the detergent industry has been reported [49,50].

The large applications of Lipases in many fields from food to medicine are due to their functionality to work in different media; these diverse applications are the reason for a huge demand in the market [51].

Different sources of lipases were reported in the past; however, the bacterial sources are more suitable and get the better chance for industrial applications [52]. Applications of lipase from microbial origin, as well as functionality in various environments, provide the ease of lipase usage in many industries. The significant part of lipase production is to introduce significant species or strain of the microbe. Thus, microorganisms play a dynamic role in lipase production. The bacterial sources have a better chance for industrial lipase production [53,54]. High GC-content bacteria within the family Pseudonocardiaceae (*Amycolatopsis eburnea*) provided a noble prospect to work on for understanding the lipase production. This genus (*Amycolatopsis*) of bacteria showed many antibiotic productions in different conditions [55,56]. Therefore, providing the lipase structural investigations beside their antibiotic properties can help in introducing them for industrial application more easily than others [57].

Recently published genetic diversity of lipase in bacteria showed great differences in lipase characterizations. However, they revealed a conserved sequence which contained penta-peptide (Gly-X-Ser-X-Gly) [52]. Seven groups (Group A-G) of bacterial lipases classified. The computational analysis of new enzymes from bacteria such as *Amycolatopsis eburnea* can provide clearer information for lipase classification and even help to introduce more clear understanding of lipase evolution. As lipases are water soluble and their substrates are mostly insoluble, their structures should dictate the specific functional activity [16]. Functional activity of this ubiquitous enzyme is very efficient in energy consuming point of view and environmental friendly in comparison to other catalyzers [58].

We should also mention that lipases are substrate specific, as well as structurally chemo-, region- and stereo- specific. Three most recognized groups of lipases can mention here as non-specific lipases, 1, 3-specific lipases and acid-specific lipases based on their catalyzing activity on triglycerides substrate in different systems. The preferred lipases for industry should have a low reaction time and remain resistant to various pH beside the activity in non-aqueous media [59]. The priority and preference of lipases with microbial origin beside the ease of their production with cheap growth media provide the better opportunity to work on their genetic manipulation towards achieving the ideal lipases [60].

In our research, the role of interacting hydrophobic residues in conserved structural regions was clearly presented. The lipases presented here clearly showed that they are homologous and the structural homology features are recognizable based on their similarity and phylogenetic dendongram. However, the need for finding structural similarity was necessary to establish a common ancestry. This structural conservation presented here clearly showed the surface plasticity in *Amycolatopsis eburnea* lipases. This structural conservation contained GLY and Val with higher percentage residues that imposed the stability and folding functionality to *Amycolatopsis eburnea* lipases. SASA features of these regions also deduced the hydrophobic contact information from the hydrophobic residues in lipase structures. The secondary structure length and loops in *Amycolatopsis eburnea* lipases were exactly related and substantially conserved. The conserved SASA could be a result of selective pressure on molecular conservation. Here, we identified 8 clusters, of which their mean of the SASA were very close. These 6 conserved regions were great tools for describing the stability and surface plasticity of lipases in different environments and their substrate specificity [61,62,63].

The finding of the role of these residues in folding or function could even be clearly answered as most of the amino acids in conserved regions are considered hydrophobic residues to some extent. Therefore, they would act in stability and folding conservation of lipases. This research could support the results and hypotheses in finding the specific residues to develop the better enzyme with mutation approaches. Furthermore, it clearly could help in sequence- structure correlation, role of individual residues in folding, stability and function.

It is important to mention here that the results clearly showed that there were specific structural constraints with specific residues SASA features conserved in *Amycolatopsis eburnea* lipases. This pattern of SASA/hydrophobic positions was observed and clearly conserved. The results showed the compensating amino acids residues that might occur during evolution were conserved in SASA features. It should be noted that amino acids mutation could be detected in conserved regions also. Thus, the only conserved feature in the conserved structure was SASA and hydrophobic features. Then the results showed the specific SASA conservation pattern to impose the native folding in homologous lipases from *Amycolatopsis eburnea*. Therefore, the significant correlation between sequences, conserved structural regions and SASA features was observed. This information could extend lipase structural information and describe the algorithms to predict SASA protein contact map in future.

Microbial lipase production needs to introduce better microbes and optimize more suitable environmental conditions. The lipase structure identification is the essential part of the system to find the better source of microbe for their production. Therefore, microbial sources can play the vital role in this selection. Traditionally many microbial sources selected for industrial production based on their amount of lipase production. However, the functionality of lipases and their efficiency can improve significantly by finding the better structure [17,50,64].

These days with the help of huge bioinformatics data in protein databases and computational analysis, finding the better lipase structure for industry is more feasible. On the other hand, as the structure of enzyme was proved to be species specific therefore working on lipases from specific species is more reasonable and practical. It should be mentioned here that working on lipase structural analysis would be a great help in finding the evolution of enzyme specially to find the essential residues to track the homology relation. On the other hand, different environments could play substantial roles in functionality of lipases. Soil usually provides the vital habitat for lipase microbe alone and in interaction with plants and other biofilm. Thus, research on lipases with soil microbe origin such as *Amycolatopsis eburnea* for industry and evolution purpose is inevitable [57].

Bacterial lipases with soil origin had shown huge diversity and variation in molecular and biochemical characterization. However, the conserved structural area such as residues related to active site (serine residue enclosed with conserved penta-peptide (Gly-X-Ser-X-Gly)) was conserved in all lipases [52]. In order to cluster the lipases many enzyme features and factors considered however the solvent accessibility of enzyme as the outstanding factor always missed. Solvent accessibility has an important impact on enzyme stability and substrate activity [65]. Even finding the hydrophobic contact area that is the opposite of SASA can provide the shed light to find the stability factor in structural evolution. In this study clearly showed the SASA of the lipases from *Amycolatopsis eburnea* had specific conserved hydrophobic contact area. This feature robustly categorized the lipases in two clusters. There was another report of lipases categorizing with other features that found seven groups, however the SASA feature didn’t considered for categorizing [66].

Furthermore, designing the new lipase as well as new primer and probe could get great help from finding the conserved SASA feature [64,67]. Purification of lipases as an important factor in industry especially for mass production could gain the benefit with conserved SASA feature too. Enzyme formulation for market and even wet lab experiments could be more approachable with knowing the conserved SASA feature [66,68].

Lipase showed the molecular mass from 19 (*Bacillus stratosphericus*) to 70 kDa [69] with activity in pH from 5 (*Pseudomonas gessardii*) to 10.8 (*Enterococcus faecium*) [70] and temperature activity from 15 (*Acinetobacter* sp. XMZ (Zheng et al. 2011) to 80 °C (*Janibacter* sp. R02) [71]. Our results showed that the range of molecular weight of *Amycolatopsis eburnea* lipases was diverse and from 24 kDa to 44 kDa however the average conserved SASA area of lipases is around 43 Å^2^.

It is important to mention that all modification of lipases from chemical modification to immobilization and UV and gamma ray irradiations as well as amino acid modification and mutagenesis need great investigate to find the effect of them on conserved SASA of lipases [72,73]. The information provided here as the SASA of *Amycolatopsis eburnea* lipases can apply as the great asset for precise engineering of lipases for agricultural and industrial purposes.

The 3-D structures of lipases with ɑ/β-hydrolase fold architecture provided here can be an outstanding tool for protein modeling and engineering the lab experiments [74,75]. The hydrolyzing fold of this enzyme was assumed to be unrelated to specific residues, however, with activity in diverse environment. As different residues are involved in the structure folding of lipases, the structural conservation and their features need to classify and investigate in more details to understand the mechanisms of lipase action. It is noteworthy to mention that parallel β-sheet of eight strands play the great role in folding structure of lipases [76].

## 5. Conclusions

It is clearly shown that lipases from *Amycolatopsis eburnea* with great impact on agricultural and industrial sectors have specific structural patterns. Therefore, for developing and designing the new lipases the substantial insight on *Amycolatopsis eburnea* lipase structure, hotspots were presented with a machine learning approach. Structural landscapes of lipases with specific conserved SASA features from *Amycolatopsis eburnea* showed the better potential to be the model to design and develop synthetic lipases with an unsupervised agglomerative hierarchical method. Finding the conserved SASA of *Amycolatopsis eburnea* lipases showed a clear need for having the specific residues with specific SASA be in the structure/sequence of the enzyme for its stability and conformation. This pattern in the enzyme structure can help in the design of the synthetic lipase and even provide a great asset to find the homology of this enzyme from an evolutionary point of view. *Amycolatopsis* bacteria with symbiotic relationship with mycorrhiza can even be good examples for soil-, bacteria- and fungi-plant interactions research, and the SASA patterns in the structure of lipase enzymes can help to investigate and understand this symbiosis in future research.

## Figures and Tables

**Table 1:** Physiochemical and SASA features of *Amycolatopsis eburnea* lipases.

Lipase	NAA	MW	pI	Asp + Glu	Arg + Lys	AI	GRAVY	TPS	TAS	TSA	SCS	BBS
1	388	40,097.38	5.75	32	28	85.90	0.096	4790.13	9644.99	14,435.12	1476	1187
2	394	41,410.78	5.29	35	29	80.84	−0.028	5317.97	9602.71	14,920.67	1488	1247
3	436	44,666.35	5.27	34	27	87.82	0.120	5554.14	10,311.73	15,865.87	1582	1279
4	404	42,150.78	5.73	38	31	90.97	0.111	5406.49	10,157.69	15,564.18	1526	1286
5	288	29,214.18	6.13	19	16	89.90	0.280	3386.73	7358.97	10,745.70	1003	793
6	252	24,997.27	4.52	24	12	97.26	0.222	3612.33	6175.37	9787.69	874	666
7	419	44,089.24	6.23	29	26	73.89	−0.097	4917.24	9462.03	14,379.28	1310	867
8	380	40,419.88	5.96	47	39	95.87	−0.110	7165.27	12,160.81	19,326.07	1719	1104

Number of amino acids (NAA), molecular weight (MW), isoelectric point (pI), total number of negatively charged residues (Asp + Glu), total number of positively charged residues (Arg + Lys), aliphatic index (AI), grand average of hydropathicity (GRAVY), Total–Polar SASA (TPS), Total Apolar SASA (TAS), Total SASA (TSA), Side Chain SASA (SCS), BackBone SASA (BBS) of lipases (SASA = Å^2^).

**Table 2:** Lipase Models properties were predicated for homology modeling and deep learning de novo. The properties for deep learning de novo are shown with *.

Lipase	Entry	Oligo State	Ligand	GMQE	QMEAN	Cβ	Solvation	Torsion	Seq Identity	Seq Similarity	Coverage	Range	QSQE	Template
1	A0A3R9KNJ9	Monomer	None	0.64	−4.02 0.69 *	−1.96 −0.48 *	−1.46 0.06 *	−3.32 0.82 *	29.49%	0.35	0.92	25–388	0.00	2veo.1.A
2	A0A3R9DUJ4	Monomer	None	0.63	−3.77 0.20 *	−1.98 −0.73 *	−0.85 −0.53 *	−3.27 0.53 *	27.22%	0.34	0.91	29–394	0.16	3zpx.1.A
3	A0A427T6P4	Monomer	None	0.56	−4.02 −0.06 *	−3.87 −2.60 *	−1.01 0.32 *	−3.14 0.36 *	26.60%	0.33	0.86	28–422	0.12	3zpx.1.A
4	A0A3R9KMI2	Monomer	None	0.63	−3.74 1.04 *	−3.15 −1.18 *	−1.82 0.26 *	−2.73 1.25 *	30.41%	0.35	0.90	22–403	0.00	3guu.1.A
5	A0A3R9EQB2	Monomer	None	0.66	−2.24 0.84 *	−1.75 −1.67 *	−2.49 −0.52 *	−1.12 1.46 *	44.80%	0.40	0.87	33–282	0.00	5h6g.1.A
6	A0A3R9F8T1	Monomer	None	0.50	−2.54 1.63 *	−2.32 −1.73 *	−1.60 −0.69 *	−1.48 2.34 *	26.39%	0.32	0.86	34–251	0.00	5h6b.1.A
7	A0A3R9DV90	Monomer	None	0.31	−5.78 −1.98 *	−3.28 −2.13 *	−3.35 −3.36 *	−4.24 −0.58 *	20.95%	0.31	0.60	99–390	0.00	4bvj.1.A
8	A0A427T2R3	Monomer	None	0.59	−4.36 1.34 *	−3.28 −0.91 *	−2.69 −0.38 *	−3.03 1.74 *	32.33%	0.35	0.87	4–378	0.00	3skv.1.A

**Table 3:** Ramachandran plot information for the 3-D structures of lipases (HM = Homology Modeling, DM = Deep learning de novo Modeling).

Lipase	Sequences	Number of Residues in Favored Region		Number of Residues in Outlier Region	
		HM (%)	DM (%)	HM (%)	DM (%)
1	A0A3R9KNJ9	90.61	95.85	3.31	1.30
2	A0A3R9DUJ4	90.93	96.68	2.47	0.77
3	A0A427T6P4	89.82	96.77	2.80	0.92
4	A0A3R9KMI2	90.79	96.02	2.89	0.25
5	A0A3R9EQB2	96.77	96.85	0.40	0.00
6	A0A3R9F8T1	93.06	98.40	2.31	0.40
7	A0A3R9DV90	88.28	91.13	4.48	1.92
8	A0A427T2R3	87.40	96.03	4.29	0.00

**Table 4:** Amino acid compositions of the *Amycolatopsis eburnea* lipases.

Lipase	Entry	Length	Ala	Arg	Asn	Asp	Cys	Gln	Glu	Gly	His	Ile	Leu	Lys	Met	Phe	Pro	Ser	Thr	Trp	Tyr	Val
1	A0A3R9KNJ9	388	62	21	4	22	4	10	10	48	5	11	37	7	2	12	33	21	26	8	16	29
2	A0A3R9DUJ4	394	58	13	10	24	4	18	11	39	4	9	34	16	4	16	30	20	29	5	18	32
3	A0A427T6P4	436	65	18	13	22	2	9	12	50	5	14	40	9	5	11	33	33	34	5	19	37
4	A0A3R9KMI2	404	69	20	8	20	4	11	18	39	9	14	38	11	3	14	28	24	21	3	17	33
5	A0A3R9EQB2	288	45	8	7	14	6	7	5	37	7	9	28	8	3	9	17	18	22	3	11	24
6	A0A3R9F8T1	252	44	9	3	10	4	13	14	31	3	4	26	3	2	0	20	11	20	3	3	29
7	A0A3R9DV90	419	51	19	13	20	4	20	9	52	8	13	31	7	7	15	25	39	27	10	19	30
8	A0A427T2R3	380	54	34	8	26	2	6	21	38	14	10	48	5	2	11	30	8	26	3	5	29

**Table 5:** The secondary structure of *Amycolatopsis eburnea* lipases sequences.

Lipase	Entry	Helix (%)	Sheet (%)	Turn (%)
1	A0A3R9KNJ9	60.8	33.0	13.1
2	A0A3R9DUJ4	62.7	37.8	13.7
3	A0A427T6P4	51.4	34.4	11.0
4	A0A3R9KMI2	68.3	50.5	12.4
5	A0A3R9EQB2	56.2	60.8	9.7
6	A0A3R9F8T1	59.9	51.6	10.7
7	A0A3R9DV90	53.7	37.7	11.5
8	A0A427T2R3	67.9	35.8	10.3

**Table 6:** Nucleus and Surface solvent accessible surface area (SASA) and average of total solvent accessible area for two environments (nucleus and surface). The data presented in angstrom (Å^2^).

Lipase	SASA	Total	Apolar	Backbone	Sidechain	Total Ave SASA
1	nucleus	1612.05	1105.63	592.96	1019.10	39.65
	surface	9087.30	6239.72	1796.53	7290.70	
2	nucleus	1535.62	975.20	514.03	1021.70	40.76
	surface	9201.62	5956.73	1673.27	7528.28	
3	nucleus	1513.82	889.97	570.11	943.72	40.16
	surface	10,211.71	6650.06	2225.90	7985.83	
4	nucleus	1897.73	1227.46	601.65	1295.97	40.74
	surface	10,134.94	6760.98	1846.70	8288.26	
5	nucleus	839.39	498.14	390.68	448.79	42.98
	surface	7280.09	5114.07	1544.76	5735.31	
6	nucleus	941.66	630.22	411.88	529.76	44.89
	surface	6561.57	4139.78	1521.13	5040.43	
7	nucleus	1295.47	805.00	517.81	777.68	49.24
	surface	9195.57	5912.66	2367.46	6828.11	
8	nucleus	1686.72	1006.49	686.57	1000.18	51.53
	surface	13,554.21	8567.14	2587.99	10,966.29	

**Table 7:** Correlation matrix of structurally conserved regions-SASA.

Correlation Matrix	Lipase 1	Lipase 2	Lipase 3	Lipase 4	Lipase 5	Lipase 6	Lipase 7	Lipase 8
Lipase 1	1.00							
Lipase 2	0.91	1.00						
Lipase 3	0.54	0.72	1.00					
Lipase 4	0.65	0.63	0.36	1.00				
Lipase 5	0.64	0.78	0.88	0.33	1.00			
Lipase 6	0.55	0.69	0.85	0.48	0.92	1.00		
Lipase 7	0.60	0.72	0.60	0.36	0.80	0.83	1.00	
Lipase 8	0.75	0.74	0.50	0.55	0.78	0.78	0.81	1.00

## Data Availability

Not applicable.

## References

[1] Balchin D., Hayer-Hartl M., Hartl F.U. (2020). Recent advances in understanding catalysis of protein folding by molecular chaperones. FEBS Lett..

[2] Dułak D., Gadzała M., Stapor K., Fabian P., Konieczny L., Roterman I. (2020). Folding with active participation of water. From Globular Proteins to Amyloids.

[3] Li J., Wang J., Zhao Y., Zhou P., Carter J., Li Z., Waigh T.A., Lu J.R., Xu H. (2020). Surfactant-like peptides: From molecular design to controllable self-assembly with applications. Coord. Chem. Rev..

[4] Zhang F., Yu L., Zhang W., Liu L., Wang C. (2021). A minireview on the perturbation effects of polar groups to direct nanoscale hydrophobic interaction and amphiphilic peptide assembly. RSC Adv..

[5] Gao J., Zheng S., Yao M., Wu P. (2021). Precise estimation of residue relative solvent accessible area from Cα atom distance matrix using a deep learning method. Bioinformatics.

[6] Konstantinidis K., Karakasiliotis I., Anagnostopoulos K., Boulougouris G.C. (2021). On the estimation of the molecular inaccessible volume and the molecular accessible surface of a ligand in protein–ligand systems. Mol. Syst. Des. Eng..

[7] Gong X., Chiricotto M., Liu X., Nordquist E., Feig M., Brooks C.L., Chen J. (2020). Accelerating the generalized born with molecular volume and solvent accessible surface area implicit solvent model using graphics processing units. J. Comput. Chem..

[8] Durham E., Dorr B., Woetzel N., Staritzbichler R., Meiler J. (2009). Solvent accessible surface area approximations for rapid and accurate protein structure prediction. J. Mol. Model..

[9] Pliego J., Mateos J.C., Rodriguez J., Valero F., Baeza M., Femat R., Camacho R., Sandoval G., Herrera-López E.J. (2015). Monitoring lipase/esterase activity by stopped flow in a sequential injection analysis system using p-nitrophenyl butyrate. Sensors.

[10] Ananthi S., Ramasubburayan R., Palavesam A., Immanuel G. (2014). Optimization and purification of lipase through solid state fermentation by *bacillus cereus* MSU as isolated from the gut of a marine fish *Sardinella longiceps*. Int. J. Pharm. Pharm. Sci..

[11] Iftikhar T., Niaz M., Ali E.A., Jabeen R., Abdullah R. (2012). Production process of extracellular lipases by *Fusarium* sp. using agricultural by products. Pak. J. Bot..

[12] Kumar A., Parihar S.S., Batra N. (2012). Enrichment, isolation and optimization of lipase-producing *Staphylococcus* sp. from oil mill waste (Oil cake). J. Exp. Sci..

[13] Ülker S., Özel A., Çolak A., Karaoğlu Ş.A. (2011). Isolation, production, and characterization of an extracellular lipase from *Trichoderma harzianum* isolated from soil. Turk. J. Biol..

[14] Laachari F., El Bergad F., Sadiki M., Sayari A., Bahafid W., Elabed S., Mohammed I., Ibnsouda S.K. (2015). Higher tolerance of a novel lipase from *Aspergillus flavus* to the presence of free fatty acids at lipid/water interface. Afr. J. Biochem. Res..

[15] Priji P., Unni K.N., Sajith S., Binod P., Benjamin S. (2015). Production, optimization, and partial purification of lipase from *Pseudomonas* sp. strain BUP 6, a novel rumen bacterium characterized from Malabari goat. Biotechnol. Appl. Biochem..

[16] Guo J., Chen C.-P., Wang S.-G., Huang X.-J. (2015). A convenient test for lipase activity in aqueous-based solutions. Enzyme Microb. Technol..

[17] Kapoor M., Gupta M.N. (2012). Lipase promiscuity and its biochemical applications. Process Biochem..

[18] Farrokh P., Yakhchali B., Asghar Karkhane A. (2014). Cloning and characterization of newly isolated lipase from *Enterobacter* sp. Bn12. Braz. J. Microbiol..

[19] Lee L.P., Karbul H.M., Citartan M., Gopinath S.C., Lakshmipriya T., Tang T.-H. (2015). Lipase-secreting *Bacillus* species in an oil-contaminated habitat: Promising strains to alleviate oil pollution. Biomed. Res. Int..

[20] Nouioui I., Carro L., García-López M., Meier-Kolthoff J.P., Woyke T., Kyrpides N.C., Pukall R., Klenk H.-P., Goodfellow M., Göker M. (2018). Genome-based taxonomic classification of the phylum *Actinobacteria*. Front. Microbiol..

[21] Ventura M., Canchaya C., Tauch A., Chandra G., Fitzgerald G.F., Chater K.F., van Sinderen D. (2007). Genomics of *Actinobacteria*: Tracing the evolutionary history of an ancient phylum. Microbiol. Mol. Biol. Rev..

[22] Bandyopadhyay D., Das K., Sen S. (2016). Exploration of extracellular phytase production by *Amycolatopsis vancoresmycina* S-12 in submerged fermentation. Int. J. Curr. Microbiol. Appl. Sci..

[23] Kshirsagar S.D., Saratale G.D., Saratale R.G., Govindwar S.P., Oh M.-K. (2016). An isolated *Amycolatopsis* sp. GDS for cellulase and xylanase production using agricultural waste biomass. J. Appl. Microbiol..

[24] Peano C., Damiano F., Forcato M., Pietrelli A., Palumbo C., Corti G., Siculella L., Fuligni F., Tagliazucchi G.M., De Benedetto G.E. (2014). Comparative genomics revealed key molecular targets to rapidly convert a reference rifamycin-producing bacterial strain into an overproducer by genetic engineering. Metab. Eng..

[25] Sharma M., Dangi P., Choudhary M. (2014). Actinomycetes: Source, identification, and their applications. Int. J. Curr. Microbiol. Appl. Sci..

[26] Kumari R., Singh P., Lal R. (2016). Genetics and genomics of the genus *Amycolatopsis*. Indian J. Microbiol..

[27] Nett M., Ikeda H., Moore B.S. (2009). Genomic basis for natural product biosynthetic diversity in the actinomycetes. Nat. Prod. Rep..

[28] Tang B., Zhao W., Zheng H., Zhuo Y., Zhang L., Zhao G.-P. (2012). Complete genome sequence of *Amycolatopsis mediterranei* S699 based on de novo assembly via a combinatorial sequencing strategy. J. Bacteriol..

[29] Verma M., Kaur J., Kumar M., Kumari K., Saxena A., Anand S., Nigam A., Ravi V., Raghuvanshi S., Khurana P. (2011). Whole genome sequence of the rifamycin B-producing strain *Amycolatopsis mediterranei* S699. J. Bacteriol..

[30] Zhao W., Zhong Y., Yuan H., Wang J., Zheng H., Wang Y., Cen X., Xu F., Bai J., Han X. (2010). Complete genome sequence of the rifamycin SV-producing *Amycolatopsis mediterranei* U32 revealed its genetic characteristics in phylogeny and metabolism. Cell Res..

[31] Damborsky J., Brezovsky J. (2014). Computational tools for designing and engineering enzymes. Curr. Opin. Chem. Biol..

[32] Sraphet S., Javadi B. (2021). Computational characterizations of GDP-mannose 4,6-dehydratase (NoeL) Rhizobial proteins. Curr. Genet..

[33] García-Guevara F., Avelar M., Ayala M., Segovia L. (2016). Computational tools applied to enzyme design—A review. Biocatalysis.

[34] Akmoussi-Toumi S., Khemili-Talbi S., Ferioune I., Kebbouche-Gana S. (2018). Purification and characterization of an organic solvent-tolerant and detergent-stable lipase from *Haloferax mediterranei* CNCMM 50101. Int. J. Biol. Macromol..

[35] Andualema B., Gessesse A. (2012). Microbial lipases and their industrial applications: Review. Biotechnology.

[36] Nema A., Patnala S.H., Mandari V., Kota S., Devarai S.K. (2019). Production and optimization of lipase using *Aspergillus niger* MTCC 872 by solid-state fermentation. Bull Natl. Res. Cent..

[37] Dutta M., Tareq A.M., Rakib A., Mahmud S., Sami S.A., Mallick J., Islam M.N., Majumder M., Uddin M.Z., Alsubaie A. (2021). Phytochemicals from *Leucas zeylanica* targeting main protease of SARS-CoV-2: Chemical profiles, molecular docking, and molecular dynamics simulations. Biology.

[38] Gasteiger E., Hoogland C., Gattiker A., Duvaud S.E., Wilkins M.R., Appel R.D., Bairoch A., Walker J.M. (2005). Protein identification and analysis tools on the ExPASy Server. The Proteomics Protocols Handbook.

[39] Yang J., Anishchenko I., Park H., Peng Z., Ovchinnikov S., Baker D. (2020). Improved protein structure prediction using predicted interresidue orientations. Proc. Natl. Acad. Sci. USA.

[40] Kumar S., Stecher G., Li M., Knyaz C., Tamura K. (2018). MEGA X: Molecular evolutionary genetics analysis across computing platforms. Mol. Biol. Evol..

[41] Kumar T.A. (2013). CFSSP: Chou and Fasman secondary structure prediction server. Wide Spectr..

[42] Fraczkiewicz R., Braun W. (1998). Exact and efficient analytical calculation of the accessible surface areas and their gradients for macromolecules. J. Comput. Chem..

[43] Rost B., Sander C. (1994). Conservation and prediction of solvent accessibility in protein families. Proteins Struct. Funct. Bioinf..

[44] Pettersen E.F., Goddard T.D., Huang C.C., Couch G.S., Greenblatt D.M., Meng E.C., Ferrin T.E. (2004). UCSF Chimera—a visualization system for exploratory research and analysis. J. Comput. Chem..

[45] Javadi B. (2020). In silico characterization of lipase architectural structure in *Rhizobium leguminosarum*. Plant Cell Biotechnol. Mol. Biol..

[46] Nadeem U., Muhammad D., Muhammad S., Özkan A., Sami U., Muhammad Q. (2015). Screening identification and characterization of lipase producing soil bacteria from Upper Dir and Mardan Khyber Pakhtunkhwa, Pakistan. Int. J. Biosci..

[47] Priji P., Sajith S., Faisal P.A., Benjamin S. (2017). *Pseudomonas* sp. BUP6 produces a thermotolerant alkaline lipase with trans-esterification efficiency in producing biodiesel. 3 Biotech.

[48] Ramos-Sánchez L.B., Cujilema-Quitio M.C., Julian-Ricardo M.C., Cordova J., Fickers P. (2015). Fungal lipase production by solid-state fermentation. J. Bioprocess. Biotech..

[49] Do H., Lee J.H., Kwon M.H., Song H.E., An J.Y., Eom S.H., Lee S.G., Kim H.J. (2013). Purification, characterization and preliminary X-ray diffraction analysis of a cold-active lipase (CpsLip) from the psychrophilic bacterium *Colwellia psychrerythraea* 34H. Acta Crystallogr. Sect. F Struct. Biol. Cryst. Commun..

[50] Yang W., He Y., Xu L., Zhang H., Yan Y. (2016). A new extracellular thermo-solvent-stable lipase from *Burkholderia ubonensis* SL-4: Identification, characterization and application for biodiesel production. J. Mol. Catal. B Enzym..

[51] Chandra P., Singh R., Arora P.K. (2020). Microbial lipases and their industrial applications: A comprehensive review. Microbial. Cell Factories.

[52] Javed S., Azeem F., Hussain S., Rasul I., Siddique M.H., Riaz M., Afzal M., Kouser A., Nadeem H. (2018). Bacterial lipases: A review on purification and characterization. Prog. Biophys. Mol. Biol..

[53] Melani N.B., Tambourgi E.B., Silveira E. (2020). Lipases: From production to applications. Sep. Purif. Rev..

[54] Uttatree S., Winayanuwattikun P., Charoenpanich J. (2010). Isolation and characterization of a novel thermophilic-organic solvent stable lipase from *Acinetobacter baylyi*. Appl. Biochem. Biotechnol..

[55] Song Z., Xu T., Wang J., Hou Y., Liu C., Liu S., Wu S. (2021). Secondary metabolites of the genus *Amycolatopsis*: Structures, bioactivities and biosynthesis. Molecules.

[56] Xing K., Liu W., Zhang Y.-J., Bian G.-K., Zhang W.-D., Tamura T., Lee J.-S., Qin S., Jiang J.-H. (2013). *Amycolatopsis jiangsuensis* sp. nov., a novel endophytic actinomycete isolated from a coastal plant in Jiangsu, China. Antonie Van Leeuwenhoek.

[57] Bharathi D., Rajalakshmi G., Komathi S. (2019). Optimization and production of lipase enzyme from bacterial strains isolated from petrol spilled soil. J. King Saud. Univ. Sci..

[58] Fjerbaek L., Christensen K.V., Norddahl B. (2009). A review of the current state of biodiesel production using enzymatic transesterification. Biotechnol. Bioeng..

[59] Street G. (1977). Handbook of Enzyme Biotechnology.

[60] Bakir Z.B., Metin K. (2016). Purification and characterization of an alkali-thermostable lipase from thermophilic *Anoxybacillus flavithermus* HBB 134. J. Microbiol. Biotechnol..

[61] Nagano N., Orengo C.A., Thornton J.M. (2002). One fold with many functions: The evolutionary relationships between TIM barrel families based on their sequences, structures and functions. J. Mol. Biol..

[62] Todd A.E., Orengo C.A., Thornton J.M. (2001). Evolution of function in protein superfamilies, from a structural perspective. J. Mol. Biol..

[63] Wierenga R. (2001). The TIM-barrel fold: A versatile framework for efficient enzymes. FEBS Lett..

[64] Leathers T.D., Rich J.O., Anderson A.M., Manitchotpisit P. (2013). Lipase production by diverse phylogenetic clades of *Aureobasidium pullulans*. Biotechnol. Lett..

[65] Mahmud S., Biswas S., Paul G.K., Mita M.A., Promi M.M., Afrose S., Hasan M.R., Zaman S., Uddin M.S., Dhama K. (2021). Plant-based phytochemical screening by targeting main protease of SARS-CoV-2 to design effective potent inhibitors. Biology.

[66] El-Fakharany E.M., Hassan M.A., Taha T.H. (2016). Production and application of extracellular laccase produced by *Fusarium oxysporum* EMT. Int. J. Agric. Biol..

[67] da Silva M.A.C., Cavalett A., Spinner A., Rosa D.C., Jasper R.B., Quecine M.C., Bonatelli M.L., Pizzirani-Kleiner A., Corção G., de Souza Lima A.O. (2013). Phylogenetic identification of marine bacteria isolated from deep-sea sediments of the eastern South Atlantic Ocean. SpringerPlus.

[68] Hassan M.A., Taha T.H., Hamad G.M., Hashem M., Alamri S., Mostafa Y.S. (2020). Biochemical characterisation and application of keratinase from *Bacillus thuringiensis* MT1 to enable valorisation of hair wastes through biosynthesis of vitamin B-complex. Int. J. Biol. Macromol..

[69] Ramani K., Kennedy L.J., Ramakrishnan M., Sekaran G. (2010). Purification, characterization and application of acidic lipase from *Pseudomonas gessardii* using beef tallow as a substrate for fats and oil hydrolysis. Process Biochem..

[70] Ramakrishnan V., Goveas L.C., Suralikerimath N., Jampani C., Halami P.M., Narayan B. (2016). Extraction and purification of lipase from *Enterococcus faecium* MTCC5695 by PEG/phosphate aqueous-two phase system (ATPS) and its biochemical characterization. Biocatal. Agric. Biotechnol..

[71] Castilla A., Panizza P., Rodríguez D., Bonino L., Díaz P., Irazoqui G., Giordano S.R. (2017). A novel thermophilic and halophilic esterase from *Janibacter* sp. R02, the first member of a new lipase family (Family XVII). Enzyme Microb. Technol..

[72] Bornscheuer U.T. (2008). Alteration of lipase properties by protein engineering methods. Oléagineux Corps Gras Lipides.

[73] Bornscheuer U.T. (2013). Enzymes in lipid modification: From classical biocatalysis with commercial enzymes to advanced protein engineering tools. Oléagineux Corps Gras Lipides.

[74] Lotti M., Alberghina L. (2007). Lipases: Molecular Structure and Function.

[75] Ollis D.L., Cheah E., Cygler M., Dijkstra B., Frolow F., Franken S.M., Harel M., Remington S.J., Silman I., Schrag J. (1992). The α/β hydrolase fold. Protein Eng. Des. Sel..

[76] Khan F.I., Lan D., Durrani R., Huan W., Zhao Z., Wang Y. (2017). The Lid Domain in Lipases: Structural and Functional Determinant of Enzymatic Properties. Front. Bioeng. Biotechnol..

